# US Adults’ Perspectives on Antibiotic Durations and Adherence to Therapy for Common Bacterial Respiratory Infections: A National Survey

**DOI:** 10.1093/ofid/ofag407

**Published:** 2026-07-21

**Authors:** Alistair Thorpe, Rachael A Lee, Julia E Szymczak, Madeline C Farrell, Angela Fagerlin, Valerie M Vaughn

**Affiliations:** Department of Population Health Sciences, Spencer Fox Eccles School of Medicine at University of Utah, Salt Lake City, Utah, USA; Department of Medicine, Division of Infectious Diseases, UAB School of Medicine, Birmingham, Alabama, USA; Department of Medicine, Division of Infectious Diseases, Birmingham VA Medical Center, Birmingham, Alabama, USA; Department of Internal Medicine, Division of Epidemiology, Spencer Fox Eccles School of Medicine at University of Utah, Salt Lake City, Utah, USA; Department of Population Health Sciences, Spencer Fox Eccles School of Medicine at University of Utah, Salt Lake City, Utah, USA; Salt Lake City VA Informatics Decision-Enhancement and Analytic Sciences (IDEAS) Center for Innovation, Salt Lake City, Utah, USA; Department of Internal Medicine, Division of General Medicine, Spencer Fox Eccles School of Medicine at University of Utah, Salt Lake City, Utah, USA

**Keywords:** antibiotic adherence, antibiotic durations, antimicrobial resistance, health communication

## Abstract

We surveyed 1475 US adults (March–April 2024) about antibiotic therapy durations and adherence. Most reported preferring longer antibiotic courses (≥7 days) and believed always finishing an antibiotic course is important. These findings highlight the need for effective strategies to align public beliefs with current recommendations supporting short duration therapy.

## Teaser

In this national survey of US adults, the authors found that most respondents preferred longer antibiotic courses than are likely necessary for common bacterial infections and endorsed the recommendation to always finish prescribed antibiotics. The authors offer five evidence-based recommendations for patient-centered communication to align preferences with current evidence on appropriate antibiotic prescribing.

Since penicillin was first used in the 1940s, clinicians and public health campaigns have consistently advised patients to “always finish the full course of antibiotics,” even if they start to feel better [1]. However, over 120 randomized controlled trials now show that, for common bacterial infections, shorter antibiotic durations are often as effective, safer, and no more likely to promote antimicrobial resistance than longer durations [2, 3]. These findings have led to growing calls to (1) *recommend shorter antibiotic durations whenever possible* [4] and (2) *stop advising patients to “always finish the full course of antibiotics”* [5].

These calls to update guidance on antibiotic use reflect important gains in evidence-based prescribing. However, while these topics have drawn considerable clinical attention [6], patients’ preferences toward different durations of antibiotic therapy and their comfort with changing guidance have been overlooked. Significant and/or unexpected changes in medical advice can confuse and frustrate patients, leading to distrust and disengagement with recommended health behaviors [7]. Revising guidance to always finish the course of antibiotics presents a significant communication challenge. Original messaging surrounding this recommendation was widely promoted, is highly memorable and intuitive, and was framed as essential for patient safety [1]. Updating guidance to favor shorter antibiotic courses also requires considering patient's beliefs about treatment effectiveness and its perceived relationship with duration. For instance, some patients favor more aggressive or prolonged medical intervention, while others prefer shorter, less invasive approaches [8–10]. In both cases, patients’ whose beliefs conflict with updated recommendations may be reluctant to adopt evidence-based practices, leading to missed clinical benefits, adverse patient outcomes, and strained patient–clinician relationships.

Developing effective strategies for communicating updated guidance on antibiotic use requires understanding how patients’ beliefs and preferences differ from current guidance. However, despite the rapid evolution of clinical evidence on appropriate durations and adherence to antibiotic therapy, little is known about how patients interpret these changes and what they believe about optimal antibiotic durations. To address this gap, we surveyed US adults to examine their beliefs about appropriate antibiotic durations and adherence and factors associated with preferences for shorter or longer courses.

## METHODS

This online survey study was deemed exempt by the University of Utah Institutional Review Board (IRB_00167676). From March 21 to April 23, 2024, respondents were recruited and compensated by Dynata, who emailed a single survey link to a pool of US adults who have agreed to be invited to take part in online survey studies. Dynata had no role in the study beyond recruitment and respondent compensation. The information page informed respondents that continuing indicated consent.

### Eligibility

Participants were eligible if they were English-reading US adults, aged ≥18 years. Eligibility criteria were determined from self-reported characteristics (Supplementary Table 2).

### Measures

Our primary outcome was self-reported course length preference, measured with a single binary item: “Which antibiotic course length would you feel most comfortable taking for a bacterial respiratory infection (eg, pneumonia)?”. Respondents selected their preference for either a short (3–5 days) or long (≥7 days) antibiotic course and were asked, “Could you tell us why you would feel most comfortable taking short/long courses? (free text).”

The rest of the survey items covered 4 general domains: demographic characteristics (eg, age, gender identity, racial/ethnic identity, US census region), health status (eg, number of comorbid conditions [11]), psychological characteristics (eg, health literacy [12], subjective numeracy [13, 14], medical maximizing [8]), and exposure to guidance on antibiotic use (eg, being told to “always finish your course of antibiotics” or “stop taking antibiotics when they feel better”; potential responses, No, Yes, I’m not sure). Survey items were derived from the literature, where previously published surveys existed. Novel questions were developed by the study team, which includes social scientists, clinicians, and health services researchers with extensive experience in antibiotic stewardship, patient communication, and online survey methodology. Survey items are shown in Table 1 and fully described in the Supplementary Data.

**Table 1.** Respondent Characteristics Overall and by Antibiotic Course Duration Preferences

	Antibiotic Duration Preference		Total (n = 1475)
Characteristics	Short Courses, 3–5 d (n = 583)	Long Courses, ≥7 d (n = 892)	
Mean age (SD)	48.3 (17.7)	54.7 (17.7)	52.2 (17.9)
Age in years—n (%)			
18–33	163 (28.0)	153 (17.2)	316 (21.4)
34–49	135 (23.2)	185 (20.7)	320 (21.7)
50–64	141 (24.2)	194 (21.7)	335 (22.7)
≥65	144 (24.7)	360 (40.4)	504 (34.2)
Gender identity—n (%)			
Male	289 (49.6)	435 (48.8)	724 (49.1)
Female	287 (49.2)	451 (50.6)	738 (50.0)
Any other identity	7 (1.2)	6 (0.7)	13 (0.9)
Racial/ethnic identity—n (%)			
Non-Hispanic White	148 (25.4)	297 (33.3)	445 (30.2)
Non-Hispanic Black	190 (32.6)	250 (28.0)	440 (29.8)
Hispanic	175 (30.0)	268 (30.0)	443 (30.0)
Any other identity	68 (11.7)	77 (8.6)	145 (9.8)
Missing	2 (0.3)	0 (0.0)	2 (0.1)
US census region—n (%)			
Northeast	97 (16.6)	158 (17.7)	255 (17.3)
Midwest	112 (19.2)	184 (20.6)	296 (20.1)
South	233 (40.0)	342 (38.3)	575 (39.0)
West	137 (23.5)	207 (23.2)	344 (23.3)
Missing	4 (0.7)	1 (0.1)	5 (0.3)
Urbanicity—n (%)			
Rural	149 (25.6)	239 (26.8)	388 (26.3)
Suburban	253 (43.4)	404 (45.3)	657 (44.5)
Urban	180 (30.9)	248 (27.8)	428 (29.0)
Missing	1 (0.2)	1 (0.1)	2 (0.1)
Educational attainment—n (%)			
High school or less	125 (21.4)	176 (19.7)	301 (20.4)
Some college or trade	194 (33.3)	293 (32.8)	487 (33.0)
≥Bachelors	263 (45.1)	422 (47.3)	685 (46.4)
Missing	1 (0.2)	1 (0.1)	2 (0.1)
Number of comorbid conditions^a^—n (%)			
0	263 (45.1)	298 (33.4)	561 (38.0)
1	125 (21.4)	244 (27.4)	369 (25.0)
2	79 (13.6)	151 (16.9)	230 (15.6)
3 or 4	76 (13.0)	147 (16.5)	223 (15.1)
≥5	40 (6.9)	52 (5.8)	92 (6.2)
Mean (SD)	1.4 (2.1)	1.5 (1.7)	1.5 (1.8)
Health literacy needs^b^—n (%)			
1–2 (never or rarely)	438 (58.8)	700 (62.8)	1138 (61.2)
3–5 (sometimes or more often)	144 (16.3)	190 (15.7)	334 (15.9)
Missing	1 (0.2)	2 (0.2)	3 (0.2)
Mean (SD)	1.8 (1.1)	1.7 (1.1)	1.7 (1.1)
Subjective numeracy^c^, Mean (SD)	4.4 (1.2)	4.6 (1.2)	4.5 (1.2)
Medical maximizing^d^—n (%)			
1 (I lean toward waiting and seeing)	90 (15.4)	127 (14.3)	217 (14.7)
2	59 (10.1)	127 (14.3)	186 (12.6)
3	148 (25.4)	173 (19.4)	321 (21.8)
4	141 (24.2)	177 (19.9)	318 (21.6)
5	83 (14.2)	142 (15.9)	225 (15.3)
6 (I lean toward taking action)	62 (10.6)	145 (16.3)	207 (14.0)
Mean (SD)	3.4 (1.5)	3.6 (1.6)	3.5 (1.6)
“I completely trust my doctor's advice about the length of antibiotic course I should take”—n (%)			
1 (strongly disagree)	9 (1.5)	10 (1.1)	19 (1.3)
2 (disagree)	18 (3.1)	7 (0.8)	25 (1.7)
3 (somewhat disagree)	35 (6.0)	26 (2.9)	61 (4.1)
4 (somewhat agree)	163 (28.0)	173 (19.4)	336 (22.8)
5 (agree)	230 (39.5)	400 (44.8)	630 (42.7)
6 (strongly agree)	128 (22.0)	276 (30.9)	404 (27.4)
Mean (SD)	4.7 (1.1)	5.0 (0.9)	4.9 (1.0)
“Even if you start to feel better, it is important to always finish a prescribed course of antibiotics”—n (%)			
1 (strongly disagree)	15 (2.6)	11 (1.2)	26 (1.8)
2 (disagree)	28 (4.8)	10 (1.1)	38 (2.6)
3 (somewhat disagree)	53 (9.1)	15 (1.7)	68 (4.6)
4 (somewhat agree)	131 (22.5)	86 (9.6)	217 (14.7)
5 (agree)	140 (24.0)	181 (20.3)	321 (21.8)
6 (strongly agree)	193 (33.1)	573 (64.2)	766 (51.9)
7 (not sure)	23 (3.9)	16 (1.8)	39 (2.6)
Mean (SD)	4.7 (1.3)	5.4 (1.0)	5.1 (1.2)
“How concerned are you about experiencing short-term side effects from taking antibiotics?”—n (%)			
1 (not at all concerned)	87 (14.9)	221 (24.8)	308 (20.9)
2 (slightly concerned)	113 (19.4)	209 (23.4)	322 (21.8)
3 (somewhat concerned)	134 (23.0)	156 (17.5)	290 (19.7)
4 (moderately concerned)	126 (21.6)	160 (17.9)	286 (19.4)
5 (extremely concerned)	106 (18.2)	118 (13.2)	224 (15.2)
6 (not sure)	16 (2.7)	28 (3.1)	44 (3.0)
Missing	1 (0.2)	0 (0.0)	1 (0.1)
Mean (SD)	3.1 (1.3)	2.7 (1.4)	2.9 (1.4)
“Have you ever heard any public health messages to ‘always finish a course of antibiotics even if you feel better’? (eg, in commercials, leaflets, or posters)”—n (%)			
No/unsure	248 (42.5)	320 (35.9)	568 (38.5)
Yes, I have heard this before	335 (57.5)	572 (64.1)	907 (61.5)
“Have you ever been told by a medical professional to ‘always finish a course of antibiotics even if you feel better’?”—n (%)			
No/unsure	188 (32.2)	160 (17.9)	348 (23.6)
Yes, I have heard this before	393 (67.4)	732 (82.1)	1125 (76.3)
Missing	2 (0.3)	0 (0.0)	2 (0.1)
Sources			
Physician (eg, primary care physician)			845/1125 (75.1)
Nurse (eg, RN, NP)			125/1125 (11.1)
Physician associate			16/1125 (1.6)
Pharmacist			207/1125 (18.4)
Non-specific healthcare (eg, ER visit)			120/1125 (10.7)
Non-healthcare source (eg, a friend)			24/1125 (2.1)
“When prescribed antibiotics for a bacterial infection, how would you feel if your clinician said that you should stop taking the antibiotics when you start to feel better?”—n (%)			
1 (very uncomfortable)	63 (10.9)	236 (26.8)	299 (20.5)
2	37 (6.4)	103 (11.7)	140 (9.6)
3	63 (10.9)	93 (10.6)	156 (10.7)
4	117 (20.3)	104 (11.8)	221 (15.2)
5	117 (20.3)	138 (15.7)	255 (17.5)
6 (very comfortable)	179 (31.1)	207 (23.5)	386 (26.5)
Mean (SD)	4.3 (1.7)	3.5 (2.0)	3.8 (1.9)
“How would you feel about the clinician who told you that you should stop taking antibiotics when you start to feel better?”—n (%)			
1 (not at all competent)	54 (9.3)	234 (26.4)	288 (19.6)
2	49 (8.4)	104 (11.8)	153 (10.4)
3	70 (12.0)	114 (12.9)	184 (12.6)
4	122 (21.0)	133 (15.0)	255 (17.4)
5	123 (21.2)	116 (13.1)	239 (16.3)
6 (very competent)	163 (28.1)	184 (20.8)	347 (23.7)
Mean (SD)	4.2 (1.6)	3.4 (1.9)	3.7 (1.8)
“Have you ever been told that you should stop taking antibiotics when you feel better?”—n (%)			
No/unsure	438 (75.1)	779 (87.3)	1217 (82.5)
Yes, I have heard this before	145 (24.9)	113 (12.7)	258 (17.5)

Respondents’ antibiotic duration preference categorized according to their response to the question “Which antibiotic course length would you feel most comfortable taking for a bacterial respiratory infection (eg, pneumonia)? Short (3–5 d) versus long (≥7 d)”.

^a^As captured by the Charlson Comorbidity Index (CCI). Maximum possible score is 12.

^b^Ability to find, understand, and use information and services to inform health-related decisions as captured by the Single Item Literacy Screener (SILS). Higher scores correspond to needing more help reading health-related materials.

^c^Individuals’ beliefs about their mathematical skills and their preferred presentation of numerical information as captured by the 3-item Subjective Numeracy Scale (SNS). Higher scores correspond to greater belief in their math skill.

^d^Preference for active versus passive approaches to healthcare as captured by the single-item maximizer–minimizer elicitation question (MM1). Higher scores correspond to preferring active versus passive approaches to healthcare in clinically uncertain scenarios.

### Analyses

Survey responses were first characterized using descriptive statistics. Second, we used 2-sided exact binomial tests to assess whether the proportion of respondents preferring long (≥7 days) versus short (3–5 days) antibiotic courses, and the proportion rating long courses as safer or more effective than shorter courses, differed from 0.50 (equal preference). Finally, as an exploratory analysis to identify characteristics associated with respondents’ preference for short (3–5 days) versus long (≥7 days) antibiotic durations, we used multivariable logistic regression. We included variables simultaneously based on conceptual relevance (demographics, health status, psychological/behavioral measures, and exposure to antibiotic-use guidance). Open-ended survey responses were reviewed for thematic content. All statistical analyses were performed with R version 4.2.2 and significance set at 0.05 (2-sided).

## RESULTS

Of the 1738 sent the survey, 1559 completed the survey (completion = 89.7%). We excluded 15 respondents who completed the survey in ≤5 minutes and 69 whose written answers led us to question the validity of their data, for a final sample of 1475. The mean reported age of respondents was 52.2 (SD = 17.9) years and 738 (50.0%) self-identified as female (Table 1).

Most respondents reported preferring longer antibiotic courses (≥7 days) over shorter ones (3–5 days) for treating a bacterial respiratory infection (60.4% vs 39.5%; binomial test, *P* < .001). Most respondents also rated longer courses as safer (61.5%; binomial test, *P* < .001) and more effective (63.0%; binomial test, *P* < .001) than shorter courses.

In open-ended responses, respondents who preferred shorter courses described having a general aversion to medication, concerns about side effects, overuse, and resistance. They also reported being more familiar with receiving shorter durations when prescribed antibiotics and a general belief that “shorter is better” for medications in general. Respondents who reported they preferred longer courses noted they were more used to them, equated longer durations with greater efficacy, and associated them as a “better safe than sorry” approach for clearing infections and preventing reinfection (see Supplementary Table 1 for example quotes).

Most respondents (88.4%) agreed that it is important to always finish a prescribed course of antibiotics, even if they start to feel better. More respondents reported receiving this advice from a medical professional (76.3%) than a public health message (61.5%; McNemar's test, *P* < .001). Among the 1125 respondents who reported receiving advice from a medical professional to always complete a prescribed course of antibiotics, the majority (75.1%) indicated that they had been told this by a physician (eg, their primary care provider). Though only 17.5% of respondents reported ever being told to stop taking antibiotics when they feel better, over half (59.2%) said they would feel at least somewhat comfortable with their clinician recommending they stop taking antibiotics when they start to feel better and would perceive the clinician as at least somewhat competent (57.4%). Trust in doctors’ advice about appropriate antibiotic durations was high (92.9%). Over half of respondents (54.3%) reported being concerned about short-term side effects from antibiotics.

**Figure 1.**
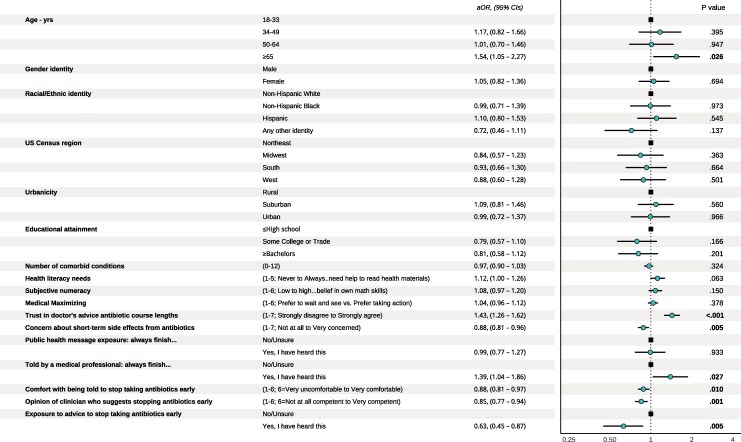
Factors associated with preferring longer antibiotic courses (≥7 d) over shorter ones (3–5 d) for treating a bacterial respiratory infection. All variables shown in the figure were included in the multivariable model. For continuous and ordinal variables, adjusted odds ratios (aORs) represent the change associated with a 1-unit increase on the corresponding scale.

On multivariable analysis, compared with those who preferred taking a short course (3–5 days), respondents who preferred longer antibiotic courses (≥7 days) were older, more trusting of their doctor's advice about antibiotic treatment durations, and more likely reported having been told by a medical professional to “always finish a course of antibiotics.” Respondents who preferred longer courses were also less worried about side effects, less likely to have been told to stop taking antibiotics when feeling better, less comfortable with being asked to do so, and perceived clinicians giving such advice as less competent (Figure 1).

## DISCUSSION

In this national online survey, we assessed how US adults interpret appropriate antibiotic durations and adherence, adding an important patient-level perspective to ongoing clinical discussions. We found that 60% of US adults expressed a preference for longer durations of antibiotic therapy than are likely necessary for common bacterial infections. This preference was related to several factors including having been told by a medical professional to “always finish a course of antibiotics” and a general belief that longer treatments are inherently better. Taken together, these findings highlight that many US adults’ antibiotic beliefs and preferences conflict with evolving clinical perspectives on appropriate antibiotic durations, the applicability of the “always finish antibiotics” mantra, and adherence plans for common bacterial infections.

Almost all respondents had heard, and still endorsed, the now-questioned guidance to “always finish the full course of antibiotics,” underscoring just how pervasive and memorable this guidance remains. Both quantitative and qualitative data indicate that the advice to “always finish the course” is a key driver of respondents’ antibiotic duration preferences. Receiving this advice from a clinician predicted preference for longer durations, and many respondents gave it as an explicit justification for preferring longer durations. Although very few respondents (17.5%) had ever been told to stop taking antibiotics when they feel better, many (58.4%) said that they would be at least somewhat comfortable hearing it from their clinician.

This study has limitations. As a cross-sectional, online, English-only survey, our data cannot be generalized to groups we were less able to reach, cannot capture change over time, and estimates from the regression do not offer unbiased population level insights. Responses were given for respiratory infections and may differ for other conditions (eg, urinary tract infections). Results also rely on self-reported attitudes and experiences which are subject to imperfect recall and social desirability biases. In addition, we presented response options as “short” (3–5 days) versus “long” (≥7 days), which may have influenced respondents’ choices in multiple ways. As reflected in respondents’ open-ended responses, these labels can carry different and sometimes competing connotations (eg, short as insufficient or quick and effective; long as excessive or thorough). The use of binary categories may also not have captured preferences for durations that fall between these anchors (eg, 6 days). Our survey questions about where respondents had received advice about antibiotic use focused on primary care physicians and may not have captured guidance from other healthcare professionals (eg, pharmacists). As a result, our findings may not fully reflect the range of messaging respondents receive across clinical and community settings.

These findings have important implications for clinicians and public health professionals aiming to inform patients about revised and updated guidance about the safety and preference for short duration therapy. Some potential evidence-based guidance to develop messages to increase alignment between patient preferences and current evidence on appropriate antibiotic prescribing include the following.

### Avoid Overly Simplistic Messaging

Although memorable and persuasive, slogan-like messaging (eg, “*always finish*” or “*shorter is better*”) can be misleading and difficult to revise as evidence evolves. Instead, emphasize that appropriate antibiotic use varies by clinical context.

### Embrace and Maintain Trust in Clinicians

Most respondents (≥90%) trusted their clinicians’ advice on antibiotic durations, an encouraging sign given trends of declining trust in science and healthcare. This finding highlights the important role of prescribers in shaping patient expectations for antibiotics. Clinicians can support alignment with current evidence on appropriate antibiotics use by clearly communicating the rationale for these decisions. Updated antibiotic guidance from trusted clinicians is likely to be well received [15], but this trust should not be taken for granted.

### Normalize Shorter Courses When Appropriate

Familiarity with longer courses was a frequently cited reason for preferring them. Increasing exposure to guideline-concordant shorter durations may help patients feel more comfortable with these recommendations.

### Highlight the Risks of Antibiotic Use

Over 40% of respondents reported little-to-no concern about side effects, which predicted preference for longer courses. Communicating risks, especially for unnecessary use, may help patients reach more informed decisions.

### Normalize Evolving Evidence

Acknowledging uncertainty and the evolving nature of scientific evidence can foster trust, understanding, and acceptance of changes [7, 16, 17]. Framing updates to antibiotic use as routine scientific progress may help patients adapt to changes.

## Supplementary Material

ofag407_Supplementary_Data

## Supplementary Data

Supplementary materials are available at *Open Forum Infectious Diseases* online. Consisting of data provided by the authors to benefit the reader, the posted materials are not copyedited and are the sole responsibility of the authors, so questions or comments should be addressed to the corresponding author.
