# Supplementary material for: US Adults’ Perspectives on Antibiotic Durations and Adherence to Therapy for Common Bacterial Respiratory Infections: A National Survey
Source: Open Forum Infect Dis. 2026 Jul 21;13(7):ofag407. doi: 10.1093/ofid/ofag407 (PMC13386500; doi:10.1093/ofid/ofag407)
Supplement: ofag407_Supplementary_Data [file ofag407_supplementary_data.docx]

**Title:** US adults’ perspectives on antibiotic durations and adherence to therapy for common bacterial respiratory infections: a national survey

**Authors:** Alistair Thorpe PhD^1^, Rachael A. Lee MD, MSPH^2,3^, Julia E. Szymczak PhD^4^, Madeline C. Farrell MSc, Angela Fagerlin PhD^1,5^, Valerie M. Vaughn MD, MSc^6^

| 1. Department of Population Health Sciences, Spencer Fox Eccles School of Medicine at University of Utah, Salt Lake City, UT, USA 2. Department of Medicine, Division of Infectious Diseases, UAB School of Medicine, AL, USA 3. Department of Medicine, Division of Infectious Diseases, Birmingham VA Medical Center, Birmingham, AL, USA 4. Department of Internal Medicine, Division of Epidemiology, Spencer Fox Eccles School of Medicine at University of Utah, Salt Lake City, UT, USA 5. Salt Lake City VA Informatics Decision-Enhancement and Analytic Sciences (IDEAS) Center for Innovation, Salt Lake City, UT, USA 6. Department of Internal Medicine, Spencer Fox Eccles School of Medicine at University of Utah, Salt Lake City, UT, US |
| --- |

**Corresponding author**: Alistair Thorpe PhD, Department of Population Health Sciences, Spencer Fox Eccles School of Medicine at University of Utah, 295 Chipeta Way, Salt Lake City, UT, USA, 84108 | (801) 587-0742 ([alistair.thorpe@hsc.utah.edu](mailto:alistair.thorpe@hsc.utah.edu))

**Supplement description**

This supplement contains the full description of survey items for the present study and supplementary tables (exemplar quotes and survey quotas).

**Survey items**

**Study information:**

We invite you to participate in a research study about decisions you make regarding your health. In this study, you will be asked some questions about your personal opinions, previous decisions, and experiences regarding antibiotics, common infections, and COVID-19. We will also ask some questions about yourself. If you agree to participate, we would like you to answer the questions on the following screens.

It will take approximately 15 to 20 minutes to complete this survey. You are free to skip any questions that you prefer not to answer. Compensation for participation will be provided in accordance with your panel agreement.

Every effort will be made to protect your privacy and confidentiality. We will not collect your name or any identifying information about you. Your participation will be completely anonymous and it will not be possible to link you to your responses.

This study is not designed to benefit you directly. You have a choice about being in this study. You do not have to be in this study if you do not want to be.

The data we collect will be used for this study but may also be important for future research. Your data may be used for future research or distributed to other researchers for future study without additional consent if information that identifies you is removed from the data.

Taking part in this research study is completely voluntary. If you do not wish to participate in this study, simply click in the corner to close the web browser window.

You may have questions about your rights as someone in this study. If you have questions, you can call the University of Utah Institutional Review Board (the responsible Institutional Review Board) at 801-581-3655. Questions or concerns about this study or interest in the final results may be directed to Dr. Valerie Vaughn at valerie.vaughn@hsc.utah.edu. Thank you for taking part in this study.

**Demographics**:

What is your age?

Skip logic: if answer is <18 then skip to End of Survey.

How would you describe your gender identity?

- Female (1)
- Male (2)
- Transgender woman/Transwoman (3)
- Transgender man/Transman (4)
- Non-Binary/Third gender (5)
- Prefer not to say (6)
- Other please specify (7)

How would you describe your ethnic group or background?

(Please select all that apply)

- American Indian or Alaskan Native (1)
- Asian or Asian American (2)
- Black or African American (3)
- Native Hawaiian or other Pacific Islander (4)
- White or European American (5)
- Other please specify (6)

Are you Hispanic or Latino/a or Latinx?

- No (1)
- Yes (2)

What state do you live in?

↓ Alabama (1)…Wyoming (52)

What is the highest level of schooling you have completed? (Check one)

- None (1)
- Elementary school (2)
- Some high school but no diploma (3)
- High school (Diploma or GED) (4)
- Some college, but no degree (5)
- Trade school (6)
- Bachelor’s degree (BS, BA, etc.) (7)
- Master’s degree (MA, MPH, etc.) (8)
- Doctoral/Professional degree (PhD, MD, etc.) (9)

How would you best describe the place where you live?

- Rural (1)
- Small city, e.g. less than 100,000 people (2)
- Suburban, near a large city (3)
- Mid-sized city, 100,000 to 1 million people (4)
- Large city, more than 1 million (5)
- Other, please specify (6)

By moving the scale point to the left or right, please indicate which antibiotic course lengths you think **are most effective** at treating bacterial respiratory infections (e.g., pneumonia)?

| Short courses (3-5 days) are most effective |  |  |  |  |  |  |  |  |  | Long courses (+7 days) are most effective |
| --- | --- | --- | --- | --- | --- | --- | --- | --- | --- | --- |
| 0 | 10 | 20 | 30 | 40 | 50 | 60 | 70 | 80 | 90 | 100 |
| *[Favors short]* | | | | *[no strong preference]* | | | *[Favors long]* | | | |

By moving the scale point to the left or right, please indicate which antibiotic course lengths you think **are safer** for treating bacterial respiratory infections (e.g., pneumonia)?

| Short courses (3-5 days) are safer |  |  |  |  |  |  |  |  |  | Long courses (+7 days) are safer |
| --- | --- | --- | --- | --- | --- | --- | --- | --- | --- | --- |
| 0 | 10 | 20 | 30 | 40 | 50 | 60 | 70 | 80 | 90 | 100 |
| *[Favors short]* | | | | *[no strong preference]* | | | *[Favors long]* | | | |

Which antibiotic course length would you feel most comfortable taking for a bacterial respiratory infection (e.g., pneumonia)?

- Short courses (3-5 days) (1)
- Long courses (7+ days) (2)

*[if 1 selected]
Could you tell us why you would feel most comfortable taking short courses? [Open text]*

*[if 2 selected]
Could you tell us why you would feel most comfortable taking long courses? [Open text]*

I completely trust my doctor’s advice about the length of antibiotic course I should take?

- Strongly disagree (1)
- Disagree (2)
- Somewhat disagree (3)
- Somewhat agree (4)
- Agree (5)
- Strongly agree (6)

Even if you start to feel better, it is important to always finish a prescribed course of antibiotics…

- Strongly disagree (1)
- Disagree (2)
- Somewhat disagree (3)
- Somewhat agree (4)
- Agree (5)
- Strongly agree (6)
- Not sure (7)

How concerned are you about experiencing short-term side effects from taking antibiotics?

- Not at all concerned (1)
- Slightly concerned (2)
- Somewhat concerned (3)
- Moderately concerned (4)
- Extremely concerned (5)
- Not sure (6)

Have you ever heard any public health messages to ‘always finish a course of antibiotics even if you feel better’? (e.g., in commercials, leaflets, or posters)?

- No, I have never heard this before (1)
- Yes, I have heard this before (2)
- I’m not sure (3)

Have you ever been told by a medical professional to ‘always finish a course of antibiotics even if you feel better’?

- No, I have never heard this before (1)
- Yes, I have heard this before (2)
- I’m not sure (3)

Have you ever been told that you should stop taking antibiotics when you feel better?

- No, I have never heard this before (1)
- Yes, I have heard this before (2)
- I’m not sure (3)

When prescribed antibiotics for a bacterial infection, how would you feel if your clinician said that you should stop taking the antibiotics when you start to feel better?

- Very uncomfortable (1), (2), (3), (4), (5), Very comfortable (6)

How would you feel about the clinician who told you that you should stop taking antibiotics when you start to feel better?

- Not at all competent (1), (2), (3), (4), (5), Very competent (6)

Now we would like to learn a little more about you. Thinking about your own personal history, experiences, and beliefs, please indicate your response to the following statements:

Sometimes, medical action is clearly necessary, and sometimes it is clearly NOT necessary. Other times, people differ in their beliefs about whether medical action is needed. In medical situations where it’s not clear, do you tend to lean towards taking action or do you lean towards waiting and seeing if action is needed? Importantly, there is no “right” way to be. Please answer on the 1-6 scale below:

- I lean toward waiting and seeing (1), (2), (3), (4), (5), I lean toward taking action (6)

As far as you know, do you have any of the following health conditions at the present time?

|  | No, I do not have  this condition (0) | Yes, I have  this condition (1) |
| --- | --- | --- |
| Asthma, emphysema, or chronic bronchitis, COPD  *(other lung disease)* (1) | ° | ° |
| Arthritis or rheumatism (2) | ° | ° |
| Cancer, diagnosed in the past 3 years (3) | ° | ° |
| Diabetes (4) | ° | ° |
| Digestive problems  *(such as ulcer, colitis, or gallbladder disease)* (5) | ° | ° |
| Heart trouble  *(such as angina, congestive heart failure, or coronary artery disease, having a past heart attack)* (6) | ° | ° |
| HIV illness or AIDS (7) | ° | ° |
| Kidney disease (8) | ° | ° |
| Liver problems (such as cirrhosis) (9) | ° | ° |
| Stroke (10) | ° | ° |
| High blood pressure (hypertension) (11) | ° | ° |
| Very overweight or obese (12) | ° | ° |
|  | | |

How often do you have someone (like a family member, friend, hospital/clinic worker or caregiver) help you read instructions, pamphlets or other written health materials from your doctor or pharmacy?

- Never (1), Rarely (2), Sometimes (3), Often (4), Always (5)

How good are you at working with fractions?

- Not good at all (1), (2), (3), (4), (5), Extremely good (6)

How good are you at figuring out how much a shirt will cost if it is 25% off?

- Not good at all (1), (2), (3), (4), (5), Extremely good (6)

How often do you find numerical information to be useful?

- Never (1), (2), (3), (4), (5), Very often (6)

**Debrief:** Thank you for your participation! You are now finished with this survey. In this study, we are interested in understanding how to communicate about the risks of antibiotic resistance and people’s attitudes and understanding of antibiotics. We greatly appreciate all your responses! We are learning a lot about these topics, and we hope to share our findings broadly soon.

For accurate, up-to-date information please see the following CDC websites:

- Antibiotic resistance: <https://www.cdc.gov/drugresistance/index.html>
- Patient resources about antibiotics: <https://www.cdc.gov/antibiotic-use/materials-references/index.html>
- COVID-19: <https://www.cdc.gov/coronavirus/2019-ncov/index.html>

Thank you for participating in this survey.

**Supplementary Tables/Figures**

| Supplementary Table 1. Exemplar respondent quotes by antibiotic duration preferences. | |
| --- | --- |
| **Reasons given for preferring shorter antibiotic courses:** | |
|  | “The least amount of time I need to be on medication is what I would feel most comfortable with. The shorter the better”  “Most medications are nasty the shorter the better.”  “I am not a fan of taking chemicals.”  “if i had to i would rather take something faster acting”  “I feel like taking it for longer is overusing it and could cause antibacterial resistance.”  “My doctor usually proscribes antibiotics for 3-5 days”  “Minimizes the adverse effects of antibiotics”  “I would feel most comfortable taking short courses because I feel like the dosage and effectiveness of the medication will be stronger and powerful.”  “The fewer days I have to take an antibiotic, the better I feel about possible side effects and the overall effectiveness of the medicine.” |
| **Reasons given for preferring longer antibiotic courses:** | |
|  | *“Most antibiotics I have ever taken were a 10 day dose and worked just fine”*  *“In the last 20 years, I haven't ever been prescribed less than 7 days! for anything”*  *“It's better to be Safe than Sorry.”*  *“When it comes to respitory issues i feel like the safer the better. Longer course will make certain that the infection is gone.”*  *“I would be more comfortable with a longer antibiotic to be sure infection is going away and would not reoccur.”*  *“It feels like it’s giving it more time to work”*  *“I would depend on my doctor’s advice, I am assuming more is better”*  *“I think it takes longer to completely get rid of bacterial infections”*  *“It works better because it spreads out the medicine over longer periods of time”* |
| *A full list of quotes can be viewed at:* [*https://rpubs.com/AlistairThorpe/PRIME-ShortVsLong_OpenText*](https://rpubs.com/AlistairThorpe/PRIME-ShortVsLong_OpenText) | |

We used recruitment quotas for self-reported age, gender identity, and racial/ethnic identity to oversample underrepresented groups as shown in Supplementary Table 2. US Census region quotas were chosen to reflect population estimates at the time of the survey according to <https://www.census.gov/topics/population.html>

| Supplementary Table 2. Survey Quotas | | |
| --- | --- | --- |
| **Characteristics** | | **Target Quotas** |
| Age in years | | |
|  | 18-33 | 22% |
|  | 34-49 | 22% |
|  | 50-64 | 22% |
|  | ≥65 | 34% |
| Gender identity | | |
|  | Male | 49% |
|  | Female | 49% |
|  | Any other identity | 2% |
| Racial/Ethnic identity | | |
|  | Non-Hispanic White | 30% |
|  | Non-Hispanic Black | 30% |
|  | Hispanic | 30% |
|  | Any other Non-Hispanic identity | 10% |
| US census region* | | |
|  | Northeast | 17% |
|  | Midwest | 21% |
|  | South | 39% |
|  | West | 24% |
|  | West | 17% |
|  | | |
